# Development of human-collaborative robots to perform daily tasks based on multimodal vital information with cybernics space

**DOI:** 10.3389/frobt.2025.1462243

**Published:** 2025-03-18

**Authors:** Akira Uehara, Hiroaki Kawamoto, Yoshiyuki Sankai

**Affiliations:** ^1^ Institute of Systems and Information Engineering, University of Tsukuba, Tsukuba, Ibaraki, Japan; ^2^ Center for Cybernics Research, University of Tsukuba, Tsukuba, Ibaraki, Japan; ^3^ R&D Center for Frontiers of MIRAI in Policy and Technology, University of Tsukuba, Tsukuba, Ibaraki, Japan; ^4^ CYBERDYNE, Inc., Tsukuba, Ibaraki, Japan

**Keywords:** cybernics, human-collaborative robot, cyborg, multimodal vital information, master-remote system, system integration

## Abstract

Due to the increasing employment of people with disabilities, support for the elderly, and childcare needs, enhancing the independence and freedom across generations and spaces is necessary. This study aimed to develop a human-collaborative robot using multimodal vital information as input with cybernics space, which is fused “human” and “Cyber/Physical Space,” and confirm its feasibility experimentally. The robot allows the user to operate it via gaze and bio-electrical signals (BES), reflecting the user’s intentions, and seamlessly transition among three modes (i.e., assistant, jockey, and ghost). In the assistant mode, the user collaborates with the robot in the physical space using a system that includes a head-mounted display (HMD) for gaze measurement, BES measurement unit, personal mobility system, and an arm–hand system. The HMD can be flipped up and down for hands-free control. The BES measurement unit captures leaked weak signals from the skin surface, indicating the user’s voluntary movement intentions, which are processed by the main unit to generate control commands for the various actuators. The personal mobility system features omni-wheels for tight turning, and the arm–hand system can handle payloads up to 500 g. In the jockey mode, the user remotely operates a small mobile base with a display and camera, moving it through the physical space. In the ghost mode, the user navigates and inputs commands in a virtual space using a smart key and remote-control device integrated with IoT and wireless communication. The switching of each control mode is estimated using the BES from the user’s upper arm, gaze direction, and position, thereby enabling movement, mobility, and manipulation without physical body movement. In basic experiments involving able-bodied participants, the macro averages of recall, precision, and F score were 1.00, 0.90, and 0.94, respectively, in the assistant mode. The macro averages of recall, precision, and F score were 0.85, 0.92, and 0.88, respectively, in the ghost mode. Therefore, the human-collaborative robot utilizing multimodal vital information has feasibility for supporting daily life tasks, contributing to a safer and more secure society by addressing various daily life challenges.

## 1 Introduction

Lifestyle and work styles are diversifying because of the increased employment of people with disabilities, support for the elderly, and childcare responsibilities. To enhance people’s independence and freedom across generations and spaces, developing robots that can be configured as a master-remote system is necessary. These robots must be capable of generating operational commands through motor and cognitive functions that many people can utilize, thereby enabling coordinated movements. For example, amyotrophic lateral sclerosis (ALS) is a progressive neuromuscular disease that causes muscle atrophy and weakness as symptoms progress (Rowland and Neil, 2001), while sensory functions such as audiovisual perception, excretion, and pain sensation and cognitive functions such as reading comprehension remain intact (Spataro et al., 2014). However, as communication through speech and writing becomes increasingly difficult, patients often experience anxiety and depression (Körner et al., 2013). Therefore, it is crucial for patients to communicate their daily needs such as movement, environmental adjustments, and nurse calls to caregivers without requiring constant third-party assistance, allowing them to live comfortably and with dignity (Wineman, 1990). Irrespective of age or disease, human-collaborative robots with cybernics space that is fused “Human” and “Cyber/Physical Space” is required to improve independence in daily life by flexibly responding to the diverse lifestyles that change based on the motor and cognitive functions and values. This robot should enable the execution of daily life tasks according to the user’s voluntary intentions, while considering temporal and spatial constraints.

Previous research and products have aimed to promote social participation in patients with neuromuscular diseases using gaze and residual finger movements to input commands or text for social networking services (OlyLab Inc, 2024) or remotely operating robot avatars for physical tasks such as serving meals and providing customer service (Barbareschi et al., 2023). Additionally, personal mobility devices that amplify slight residual movements (Imahori et al., 2019) or integrate environmental recognition and vital sensing have been developed to support elderly individuals and patients in moving as they wish (Sankai et al., 2020). However, these activities were conducted in either physical space or cyberspaces. To improve the independence of elderly individuals and neuromuscular patients, it is desirable to transit seamlessly between physical space and cyberspace, allowing them to live their daily lives in a hybrid space similar to that of the real world. Communication relies on interpreting slight eye movements or facial expressions for families and caregivers of people with ALS or prolonged consciousness disorders (Hanson et al., 2011). Therefore, interfaces must balance the acquisition of user intentions and presentation of information without covering the patient’s face. While a see-through head-mounted display (HMD) (Nonoka et al., 2024) and interfaces that display the face through a tablet (Faridan et al., 2023) have been developed, they face difficulties in directly reading subtle facial expressions and require constant device placement on the face. For daily use by elderly individuals and patients, these devices must enhance immersive experiences, reduce viewing fatigue, and improve the visibility of the patient’s face to third parties. Although cybernic interfaces using bioelectrical signals (BES) and gazes to convey movement intentions and send commands from the cyberspace to IoT devices in the physical space have been developed (Uehara and Sankai, 2024), the interface is limited to tasks that IoT devices are capable of handling. Moreover, the interface is not able to control the object with continuous minor adjustments because the flow from the user’s intention to the execution of the desired action is one-directional. For these reasons, it has difficulty in supporting daily activities, such as moving around at living spaces or manipulating household items. Therefore, it is essential to enable user interaction with human-cooperative robots that can assist movement and manipulation in the physical space based on the cybernic interface capable of transferring BES and gaze that reflect the user’s movement intentions.

For enhancing the independence and freedom across generations and spaces, this study aimed to develop a human-collaborative robot using multimodal vital information as input that reflected the human’s intention with cybernics space and confirm the feasibility of performing daily life activities through a basic experiment.

## 2 Materials and methods

### 2.1 System configuration

To improve independence and quality of life, it is necessary for elderly individuals and patients to perform daily living tasks without temporal and spatial constraints using a cybernic interface that can connect the central nervous system to cyberspace and physical space. Although the elderly individuals and patients gradually experience deterioration in motor function due to aging and the progression of symptoms and become unable to move their bodies, their eye movement function remains till relatively late in life. Moreover, BES that leak from the skin surface when the musculoskeletal system moves according to motor commands from the central nervous system can be observed even when the body is barely moving, generating an interactive bio-feedback loop between an individual’s nervous system and the worn cyborg hybrid assistive limb (HAL) based on BES applied for neuromuscular diseases, including ALS (Morioka et al., 2022). For these reasons, some products using eye gaze or BES have been used for the communication of patients with neuromuscular diseases, and gaze and BES as multimodal inputs owing to their robustness will be appropriate for patients’ input about mobility and dialog.

**FIGURE 1:**
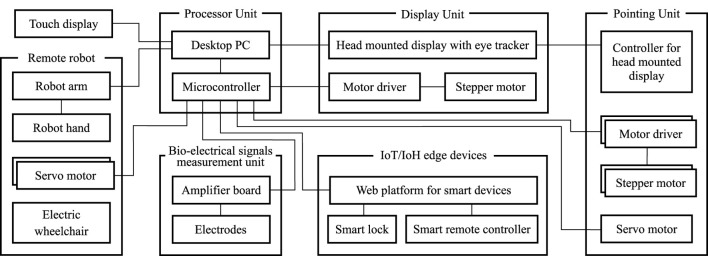
System configuration of the proposed human-cooperative robot.

Figure 1 illustrates the system configuration of the proposed human-collaborative robot. The robot consists of a processor unit, a BES measurement unit, a display unit, a pointing unit, a remote robot, IoT/IoH edge devices, and a touch display. The processor unit consists of a desktop PC and a microcontroller for communication between each unit and the device. This unit processes the measured gaze and BES signals and generates a control command. The BES measurement unit consists of an electric circuit and disposable electrodes that reflect the user’s intentions in the robot’s coordinated movements with minimal physical movement. This unit is configured to measure weak electrical signals emitted from the skin surface that reflect the user’s voluntary movement intentions through multiple electrodes. The display unit consists of a video-see-through HMD with eye-trackers and a stepper motor that controls the up-and-down flipping of the HMD to enable users to freely navigate between physical and virtual spaces. This unit was designed to drive a link mechanism, allowing the HMD to facilitate hands-free attachment and detachment. The motor used for flipping up and down was controlled by triggering the BES. The pointing unit consists of a controller for the HMD to select the object in the virtual space, with one servo motor used to click the button of the controller and two stepper motors used to rotate the pan and tilt. The remote robot consists of a robot arm, a robot hand, an electric wheelchair, and two servo motors to control the joystick of the electric wheelchair based on gaze input. To manipulate everyday items, an arm–hand system with a payload capacity of 500 g was used because 92% of the objects grasped by humans have a mass of 500 g or less (Feix et al., 2014). Considering movement in confined spaces, such as daily living environments, a personal mobility system with omni-wheels was employed as an electric wheelchair for high maneuverability. The IoT/IoH edge devices can perform simple tasks using cloud platform and API. The user could see the contents when the HMD was flipped up, check the detailed system condition, and operate each unit through the touch display.

### 2.2 UX design

To enhance independence in daily life, it is essential to develop an interface that allows users to switch input–output configurations based on the characteristics of the tasks they wish to perform and their environment. While the developed cybernic interface (Uehara and Sankai, 2024) is effective for daily tasks that can be managed by IoT devices, tasks requiring dynamic interactions, such as movement or manipulation in the physical space, necessitate a master-remote system between the user and the robot. For daily tasks involving dynamic interactions that occur in close proximity to the user, conventional cybernics technology, which enables the control of a wearable cyborg HAL (Morioka et al., 2022) or ride-on robot (Sankai et al., 2020) based on the user’s movement intentions and physiological state, is effective. Conversely, if the task location is physically distant from the user, remote operation technology (Barbareschi et al., 2023) allows the control of robots positioned at the task location by use of the user’s remaining motor functions, proving more effective in such scenarios. For these reasons, the human-collaborative robot performed the daily life activities required by the user as a master-remote system by generating operation inputs and seamlessly transitioning between the three modes using gaze and BES as inputs. These signals reflect the user’s intentions. In the assistant mode, the user collaborates with the robot in the physical space, enabling interactions that involve force feedback, such as moving objects. In the jockey mode, the user remotely operates a robot situated at a distant location, facilitating interactions that solely rely on audiovisual information, including conversations and visual inspections. In the ghost mode, the user inputs commands to IoH/IoT devices within the physical space, such as turning a television or power switch on and off or navigating within a digital twin space through the virtual space. The movement in all directions and the manipulation of objects are performed through the command input rather than the text input to reduce the burden on the user. Table 1 lists the characteristics of the task to be performed and the relationship between the environment and each mode. In the assistant mode, the user operates a ride-on robot capable of both movement and manipulation. Although this mode is challenging for navigating steps and narrow spaces, it can handle a wide range of tasks in the physical space. In the jockey mode, the user controls a mobile robot equipped with audiovisual capabilities. Although it cannot perform tasks requiring force feedback in the physical space, it is highly maneuverable. In the ghost mode, the user sends instructions to the physical space from a virtual space. This mode limits the range of tasks executable in physical space but is less constrained by time and space. The three modes were switched based on the thresholds of gaze and BES. The thresholds of gaze were set as some areas of a two-dimensional plane. The thresholds of BES were set on comparison with the amplitude of the static and dynamic conditions (Uehara and Sankai, 2024).

**TABLE 1:** Characteristics for each operation mode.

	Movable range	Task specification	Degree of freedom for task
Assistant mode	Wheelchair	Force, vison, hearing, and auditory	Physical interaction
Jockey mode	Mobile robot	Vision, hearing, and auditory	Observation
Ghost mode	Virtual space	Vision, hearing, and auditory	Simple command

### 2.3 System operation

**FIGURE 2:**
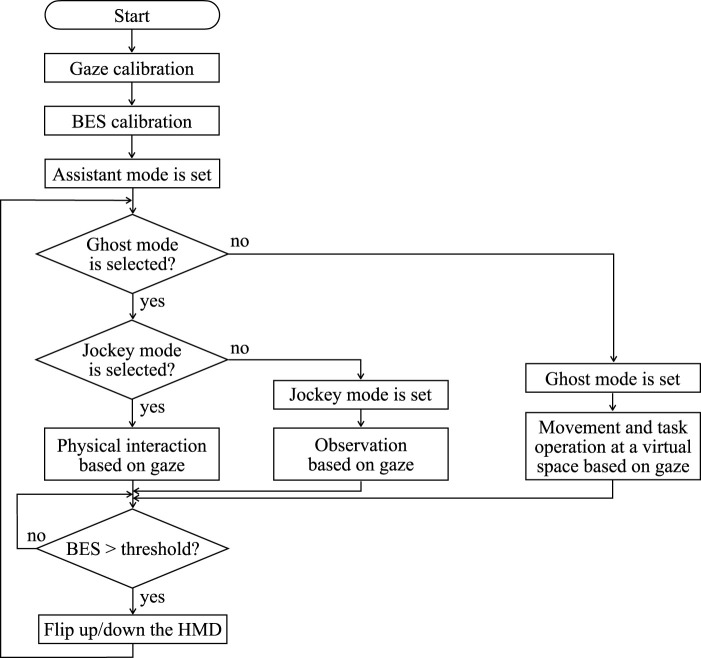
Flowchart of system operation for the proposed human-cooperative robot.

Figure 2 shows a flowchart of the system operation of the proposed human-collaborative robot. First, the user sets individual parameters for gaze and BES in each calibration phase. After calibration, the assistant mode is set as the initial condition, and the space transition, remote robot, and IoT/IoH edge devices are set using gaze and BES. When the user requires virtual mobility or operates tasks and dialogs in cyberspace, the HMD is flipped down for close contact with the face, and the user operates the HMD application using gaze and BES. Conversely, when the user does not require virtual interaction, alternating real but face-to-face interaction without some devices, the HMD is flipped up to directly see the user’s face, and the user communicates in a conventional way, as before, using eye movements and facial color grasped by the patients’ family members and caregivers. The range of motion of the motor was determined in advance based on the positional relationship between the user and the robot.

In the assistant and jockey modes, the robot consists of a processor unit, a BES measurement unit, a display unit, a remote robot, and a touch display. In the ghost mode, the robot consists of a processor unit, a BES measurement unit, a display unit, a pointing unit, and IoT/IoH edge devices. Switching between HMD attachment and detachment was controlled by estimating commands from the BES measured from the user’s skin surface. The commands for controlling the movement and rotation of the mobile base, navigating in the virtual space, and performing reaching and grasping tasks with the arm–hand system were estimated from the user’s gaze. When using the HMD, a pass-through function allows the user to recognize the position and orientation of the arm–hand system and the surrounding environment.

### 2.4 Proof-of-concept model

**FIGURE 3:**
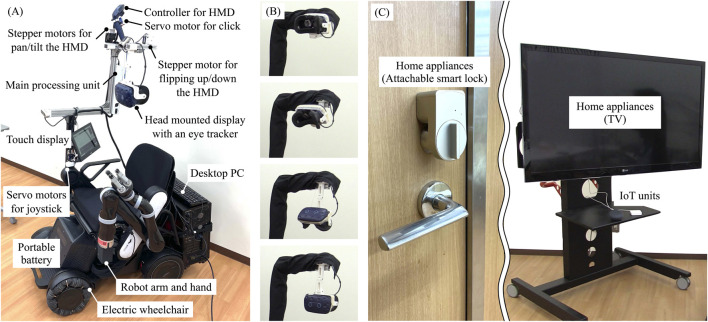
**(A)** System of proof of concept for the human-cooperative robot. **(B)** Sequential photographs of flipped up and down the HMD. **(C)** Overview of IoT devices.

**FIGURE 4:**
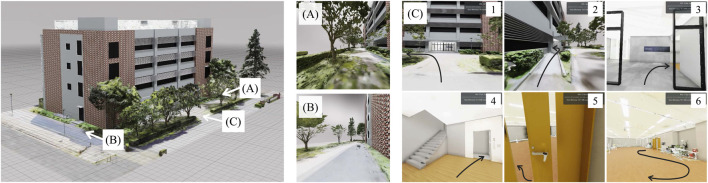
Virtual environment for the ghost mode. **(A)** Perspective of position A. **(B)** Perspective of position B. **(C)** Sequential perspectives of turning left from the front of the building, passing through the ramp, passing through the automatic door and turning right, boarding the elevator and moving to the second floor, passing through the manual door and turning left, and moving around inside the room.

Figure 3A shows the developed proof-of-concept model for a human-collaborative robot to perform daily living tasks independently. The developed robot consists of WHILL C (WHILL Inc., Tokyo, Japan) for personal mobility, KINOVA Gen 2 (Kinova Inc., Quebec, Canada) as the robot arm and robot hand, and Vive Pro Eye (HTC Corporation, New Taipei, Taiwan) as a video-see-through HMD with an eye-tracker. In the system, an HMD application for displaying the virtual environment was built with NVIDIA Omniverse (NVIDIA Corporation, California, United States) for high immersion through fast rendering and large-scale integration with avatars and IoT/IoH edge devices; the web platform was Google Assistant (Alphabet Inc., California, United States), and the IoT devices were Nature Remo (Nature Inc., Kanagawa, Japan) as the smart remote control and Open SESAME (CANDY HOUSE Inc., California, United States) as a smart lock. Figure 3B shows sequential photographs of the HMD being flipping up and down the HMD by link mechanism, pulley, wire, and stepper motor. Figure 3C presents the IoT devices of the remote controller for the TV and the lock for the door. Figure 4 shows the virtual environment of the ghost mode. The space was constructed by measuring the Center for Cybernics Research Building at the University of Tsukuba using light detection and ranging (LiDAR) and cameras. At the developed bio-electrical measurement unit, the sampling rate of BES was 1 kHz, and the signal was smoothed by 100-points moving average (Uehara and Sankai, 2024).

### 2.5 Basic experiments

The jockey mode consists of the function of selecting movement commands in the assistant mode, and the command movement functions in the front–back and left–right directions in the ghost mode. Through basic experiments in the assistant mode, in which the user cooperates with the robot in physical space, and the ghost mode, in which the user moves and inputs commands in virtual space, this study confirms the applicability of a human-collaborative robot for performing multimodal, biometric-based daily life actions. The experiment confirms the applicability of multimodal biometric-based daily life activities using a human-collaborative robot. The tasks performed in each experiment were set as use cases considering daily life actions. The experiment was conducted five times for each of the following two types of tasks using the assistant and ghost modes. The electrode for BES measurement was attached at the right upper arm.

#### 2.5.1 Experimental evaluation

The primary endpoint was the success rate of the task, and the secondary endpoints were the execution time of each task and score of the system usability scale (Tamantini et al., 2023). The initial state of the participants in the virtual room involved sitting on a chair. The participants were two able-bodied individuals. This study was approved by the Research Ethics Committee of the Graduate School of Systems and Information Engineering, University of Tsukuba. Informed consent was obtained from all participants. All experiments were conducted according to the tenets of the Declaration of Helsinki.

#### 2.5.2 The assistant mode: co-operative behavior in physical space

**FIGURE 5:**
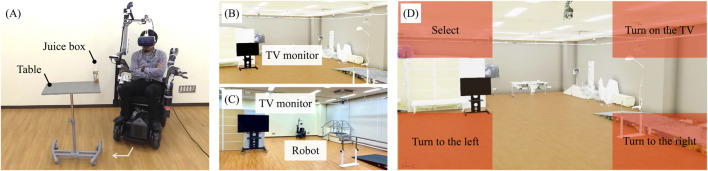
**(A)** Environment for the basic experiment. **(B)** Virtual environment for the ghost mode. **(C)** Actual environment for the ghost mode. **(D)** UI for the gaze input at the virtual space.

Figure 5A shows the environment of the experiment in the assistant mode. The tasks performed as co-operative behaviors in the physical space involved approaching and handling a drink. The drink to be handled weighed 250 g and was placed on a table at a height of 1 m. The table was placed at a position 0.3 m from the front wheels of the robot, turning 45° clockwise and a further 0.3 m away from that position. The task consisted of the following actions:

1. Move 0.3 m straight ahead.
2. Turn 45° clockwise.
3. Move 0.3 m straight ahead.
4. Grasp the target drink by moving the robot arm’s hand tip to grasp it and bring it to the mouth.
5. Remove the HMD.

#### 2.5.3 The ghost mode: Moving in the virtual space and inputting commands

Figure 5B illustrates the virtual environment of the experiment for the ghost mode, which is based on the real world presented in Figure 5C. Figure 5D illustrates the UI at the virtual space by the gaze. The task performed as movement and command inputs in the virtual space was the on/off operation of the television. The TV to be operated was positioned at a 30° counterclockwise angle in the virtual space and in front of the user in the physical space. The task consisted of the following actions:

1. Counterclockwise swimming to 30°.
2. Selecting TV.
3. Commanding it to turn on the TV.

## 3 Results

Tables 2, 3 present the experimental results for the relationship between the participant’s input and the robot’s output. In the assistant mode, the macro averages of recall, precision, and F score were 1.00, 0.90, and 0.94, respectively. In the ghost mode, the macro averages of recall, precision, and F score were 0.85, 0.92, and 0.88, respectively. The duration times were 56.3 ± 10.4 s and 27.2 ± 9.4 s, and the SUS score was 68.75 ± 1.25. In the assistant mode, the number of errors was two times for not grasping the target drink and two times for not flipping up the HMD. In the ghost mode, the number of errors was two times of “Go straight” and one time of “Select” when the participant operated “Turn to the left,” and two times of “Turn to the right” when the participant operated “Action.”

**TABLE 2:** Experimental results for the assistant mode.

Label	Recall	Precision	F score
Turn to the right	1.00	1.00	1.00
Go straight	1.00	1.00	1.00
Manipulation	1.00	0.80	0.89
Flipping up the HMD	1.00	0.80	0.89
Macro average	1.00	0.90	0.94

**TABLE 3:** Experimental results for the ghost mode.

Label	Recall	Precision	F score
Turn to the left	0.87	1.00	0.93
Action	0.83	0.83	0.83
Macro average	0.85	0.90	0.94

## 4 Discussion

### 4.1 Feasibility for supporting daily living tasks by its own

All the performance metrics about “Action” in the ghost mode have the potential to be improved by adjusting the UX to real-time drawing of the user’s gaze position at the display and gaze target area such as width, height, and position to reduce input errors, though usability was acceptable considering the SUS score. The experimental results show that the participants were able to almost manipulate the daily objects and operate home appliances independently by BES and gaze while not moving their own bodies, similar to elderly individuals and patients. The participants performed the tasks through a seamless transition between cyberspace and physical spaces using a human-collaborative robot, despite some unnecessary additional inputs. These results indicate that the proposed human-collaborative robot operated by the remaining voluntary motor functions of the users can improve independence with enough usability. Therefore, the developed robot has feasibility for performing daily living tasks using the proposed human-collaborative robot. The improvement in independence not only enhances dignity but also leads to a reduction in caregivers’ burden and medical expenses.

The reason of errors for manipulation in the assistant mode was difficulty in approaching the appropriate position of the end point of the arm–hand system when the user was not able to recognize the environment and the arm–hand position and posture using view of pass-through HMD. To reduce these errors, it is effective to incorporate a multi-camera viewpoint switching function and automate the control of reaching and handling for the target object partially. Moreover, the periodical fast calibration function about BES and gaze can reduce the false-detection and non-detection errors about HMD attachment or detachment operation and gaze input. In this study, the proof-of-concept robot was designed for reducing the mis-predicted error rather than improving the response time to prevent a decline in UX.

To balance between preventing the decline of UX and improving response time, adjusting the threshold value with an emphasis on accuracy is necessary to reduce mis-predicted errors in input by gaze because the input method has a trade-off between speed and accuracy. Additionally, the robot can be expanded to various diseases and severities by adjusting the measurement area and sensitivity of each piece of biological information regarding the multimodal input. The proposed system has potential for specific optimization of individuals by considering the user’s condition and environment by reconstructing I/O configurations utilizing other assistive robots and neuro-machine interfaces that can be used only in the physical space or cyberspace. Moreover, the system will integrate the AI processing, such as compensating the lack of data (Cheng et al., 2024) and specific optimization of individuals (Ziyu et al., 2024), to improve the reliability of user’s input that reflects the intention effected by physical or neurological dysfunction.

### 4.2 Limitations

This study showed only the basic performance of the developed robot for a proof-of-concept with able-bodied individuals. Therefore, it is necessary for various users to perform the daily living tasks in a real environment by increasing the types of commands for autonomous control, increasing the degrees of freedom through voluntary control of movement and manipulation, and automatically adjusting parameters and thresholds. The minor adjustment of the angle of rotation and the amount of movement is difficult for the participants because the developed robot generated the control command for robot operation and virtual mobility by comparing the position and time with the threshold values of gaze with a constant parameter. Thus, it is essential to improve the usability by improving the resolution of multimodal vital input such as gaze (Nonoka et al., 2024) or pupil diameter (Nonoka et al., 2025) and combining text input functions. Constructing a virtual environment that considers the real world is limited and difficult because of professional challenges and huge processes based on 3D scanning and photogrammetry. 3D environment construction by a robot utilizing NeRF (Shen et al., 2024) is useful for adaptation to any cyberspace.

## 5 Conclusion

This study presented a human-collaborative robot using BES and gaze as multimodal vital inputs generated by the remaining motor functions of users to improve independence among elderly individuals and neuromuscular patients. In a basic experiment with two able-bodied participants, they were able to operate the robot without moving their bodies, which was similar to elderly individuals and neuromuscular patients. Therefore, the developed human-collaborative robot utilizing the user’s intention has feasibility for performing daily living tasks to improve independence with enough usability. The human-collaborative robot with cybernics space that can connect the central nervous system to cyberspace and the physical space can potentially remove constraints due to motor dysfunctions and spatial limitations. To reduce the burden, differences among individuals, and frequency of human error at selecting objects or sending commands, future works will be devoted to integrating the function of object recognition based on machine learning and autonomous control by using depth information in the developed robot.

## Data Availability

The raw data supporting the conclusions of this article will be made available by the authors, without undue reservation.
